# A Non-Synonymous Single Nucleotide Polymorphism in an *OPRM1* Splice Variant Is Associated with Fentanyl-Induced Emesis in Women Undergoing Minor Gynaecological Surgery

**DOI:** 10.1371/journal.pone.0048416

**Published:** 2012-11-07

**Authors:** Grace Su Yin Pang, Farida Ithnin, Yin Yee Wong, Jing Bo Wang, Yvonne Lim, Alex Tiong Heng Sia, Caroline Guat Lay Lee

**Affiliations:** 1 Liver Cancer Functional Genomics Laboratory, National Cancer Centre Singapore, Singapore, Singapore; 2 Department of Palliative Medicine, National Cancer Centre Singapore, Singapore, Singapore; 3 Department of Biochemistry, Yong Loo Lin School of Medicine, National University of Singapore, Singapore, Singapore; 4 DUKE-NUS Graduate Medical School, Singapore, Singapore; 5 Department of Obstetric Anaesthesia, KK Women's and Children's Hospital, Singapore, Singapore; University of Washington, United States of America

## Abstract

**Background:**

Fentanyl-induced emesis (FIE) is a distressing adverse effect in the postoperative setting. The genetic basis of FIE remains largely unknown, therefore, we examined whether it was associated with specific genetic variants of *OPRM1*, the gene encoding the main receptor target of fentanyl.

**Methods:**

In this prospective case-control study, 193 women undergoing gynaecological surgery under a standardized anaesthetic with a low emetogenic risk were enrolled. Inclusion and exclusion criteria were designed to select extreme phenotypes as well as to ensure that most major confounders for FIE were either excluded or present in all patients. To control for unforeseen intra- and postoperative confounders for FIE, only 161 patients were further analysed, out of which 10 were categorized as having FIE, defined by the presence of at least one of three symptoms: nausea, vomiting or retching that was likely to be fentanyl-related. To identify SNPs relevant to FIE in our population, DNA from 40 controls and 10 cases was sequenced at the following *OPRM1* regions: 3 kbp of the promoter, main and alternative exons as well as 2 kbp of the 3′ downstream region. The genotype of the significant SNP was further determined in the remaining 111 controls.

**Results:**

The incidence of FIE was 6.2%. Initial sequencing of 10 cases and 40 controls identified 25 SNPs. Only rs540825, a non-synonymous SNP in the splice variant, MOR1X, showed a significant association with FIE post-Bonferroni correction. This SNP was further examined in the remaining 111 controls which confirmed its significant association with FIE (p = 0.019 post-Bonferroni, OR: 5.6, 95% CI: 1.42–21.91).

**Conclusions:**

This is the first report of an association between the occurrence of FIE in Chinese women undergoing gynaecological surgery and an *OPRM1* splice variant SNP, rs540825.

## Introduction

Emesis is a distressing side effect of administering opioids such as fentanyl resulting in adverse consequences [1], [2] but not all patients who are administered opioids develop emesis. Genetic variants are one possible explanation for inter-individual differences in FIE occurrence [3], [4].

Single nucleotide polymorphisms (SNPs) are the commonest variant in the human genome [5] so SNPs are obvious potential candidates for initial study when identifying genetic variants predisposing to FIE. The mu-opioid receptor (MOR), encoded by the gene, *OPRM1*, is the main receptor target for fentanyl. We hypothesized that SNPs in *OPRM1* could explain some of the susceptibility to FIE.

Although SNPs in *OPRM1* can affect *OPRM1* expression and function *in vitro* [6], [7], establishing an association between *OPRM1* genetic variants and opioid-induced emesis (OIE) have been less successful [8]. Potential reasons for studies not identifying an association between OIE and any genetic variant include heterogeneity in individuals included in such studies (e.g. mixed ethnic groups), variability of opioids and route of administration, and the concomitant use of non-opioid emetogenic drugs and/or anti-emetics resulting in too many confounders to allow identification of a specific phenotype.

To address the issue of study heterogeneity, the patients' eligibility criteria as well as the study protocol in our case-control study was designed so that all study subjects were similar with respect to confounders of OIE including previously reported major non-opioid emetic risk factors [9], [10] such as gender as well as motion sickness to allow us to ascertain if the emesis observed was likely to be due to the opioid.

Patients receiving the anaesthetic regime in this study were not expected to develop postoperative emesis. Therefore, patients who developed postoperative emesis could be considered to represent the extreme upper end of the emetic risk spectrum and thus regarded as extreme phenotypes. Extreme phenotypes for adverse drug reactions (ADRs) are known to demonstrate a strong genetic basis [11]. Hence, studying extreme phenotypes in OIE is a useful strategy for us to elucidate the genetic basis of OIE.

The large genetic effect size observed in extreme phenotypes is advantageous as study power can be maintained with a smaller study sample size [12], [13]. For example, Nelson et al demonstrated, in a genomewide association study (GWAS) of 500000 SNPs and abacavir hypersensitivity, that only 14 cases and 200 clinically matched controls were needed to attain 80% statistical power to detect a significant association (p-value<10^−7^) between an adverse drug reaction (ADR) with 5% prevalence and SNP with minor allele frequency (MAF) of 5% but large genetic effect of 30 for a dominant model [14].

In contrast to previous studies on OIE, which genotyped a few selected SNPs in OPRM1, we elected to sequence functionally important genomic regions of *OPRM1* [8], [15]. Sequencing was performed to identify novel SNPs and avoided the *a priori* assumption made in genotyping; that the genotyped SNP was the causative SNP. Sequencing extreme phenotypes has been reported to be a powerful strategy for discovering SNPs associated with complex phenotypes [16], [17] which OIE is likely to be.

*OPRM1* SNPs significantly associated with OIE could be used as a starting platform for further studies on genetic markers not only for OIE but also postoperative nausea and vomiting (PONV).

## Materials and Methods

Approval was obtained from the National Cancer Centre Singapore (NCCS) and the KK Women's Hospital (KKWH) ethical review committees (CRIB 2005/427/B) before commencing our case-control candidate gene study. Written informed consent was obtained from the patient before enrolment into the study.

### Preoperative Patient Selection Criteria

Our study inclusion and exclusion criteria (Table 1) was designed to ensure that all study subjects were similar with respect to previously reported non-opioid emetic risk factors such as female gender and history of motion sickness [9], [10]. We did not verify whether patients had a history of prior postoperative PONV as this information is subject to recall bias and a diagnosis of PONV does not imply that emesis is necessarily opioid-related.

**Table 1:** Inclusion and exclusion criteria for study subjects.

INCLUSION	EXCLUSION
Females aged 18–80 years	Declined to participate
Subjects parents and grandparents are of Chinese descent by self-report	Allergic to opioids
American Society of Anaesthesiologists (ASA) physical status I and II	Undergoing termination of pregnancy or laparoscopic surgery
Undergoing gynaecological day surgery at the Day Surgery Unit of KKWH	
Intact cognition	
Body mass index (BMI)<35	
No history of emesis or anti-emetic usage for at least 2 days prior to entry into the study	
Opioid-naïve	
Non-smokers	
Not pregnant	
No history of migraine or motion sickness	
No history of alcohol or drug abuse	

Abbreviations: KKWH: KK Womens Hospital.

### Perioperative Protocol

**Figure 1:**
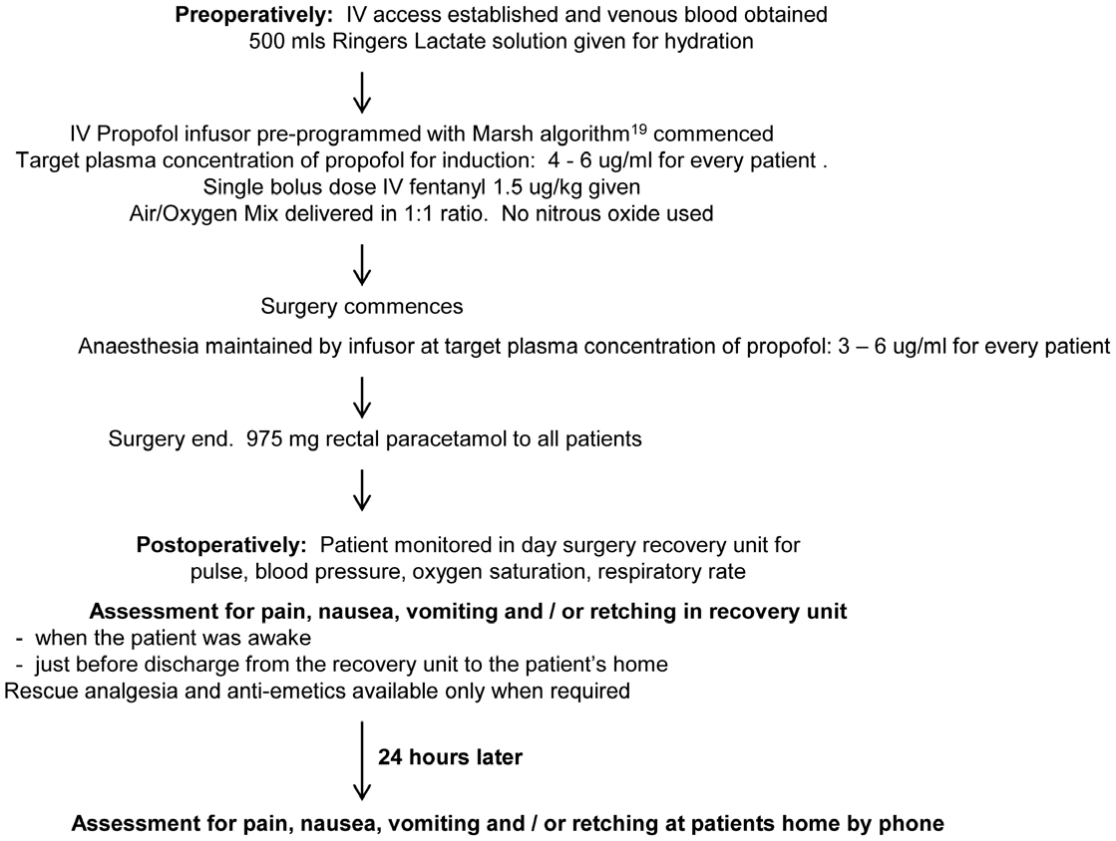
Flowchart of study perioperative anaesthetic protocol of low emetogenicity for minor gynaecological day surgery. No prophylactic anti-emetics were given pre and intraoperatively. Total intravenous anaesthesia (TIVA) using an intravenous (IV) target controlled infusion (TCI) of propofol, was administered using a propofol infusor (Asena PK Syringe Pump, Carefusion USA). The infusor was pre-programmed with the Marsh algorithm [18], which computed propofol dosing rates to attain a target perioperative plasma propofol concentration of 4–6 ug/ml and 3–6 ug/ml in the induction and maintenance phase of anaesthesia respectively in every patient.

The perioperative protocol has been outlined as a flowchart in Figure 1. Propofol-based Total Intravenous Anaesthesia (TIVA) without nitrous oxide was administered. To attain a perioperative plasma propofol concentration of 4–6 ug/ml and 3–6 ug/ml during the induction and maintenance phase of anaesthesia respectively, the Marsh algorithm [18], pre-programmed in the propofol infusor (Asena PK Syringe Pump, Carefusion USA), was employed to compute the propofol dosing rates based on the weight, age and gender of each patient. Hence, the total propofol dose administered was different amongst different patients.

Intravenous (IV) administration of a single bolus dose of 1.5 ug fentanyl/kg body weight, which is a highly selective MOR agonist with no active metabolites, was used to preclude the possibility of differences in emetic occurrence due to differences in types of opioids [19], their active metabolites or routes of administration, The drug was administered at induction with no further doses given intraoperatively.

The only additional analgesic allowed by protocol was paracetamol. No other opioid or non-opioid analgesics such as non-steroidal anti-inflammatory drugs (NSAID), or anti-emetics were given pre or intraoperatively.

Postoperatively, patients were assessed for the presence or absence of pain, nausea, vomiting and retching at 3 time points; when the patient was awake, just before discharge from the day surgery unit and the next day via telephone to the patient. Analgesics and anti-emetics were available as rescue medications only when required.

### Analyses

**Figure 2:**
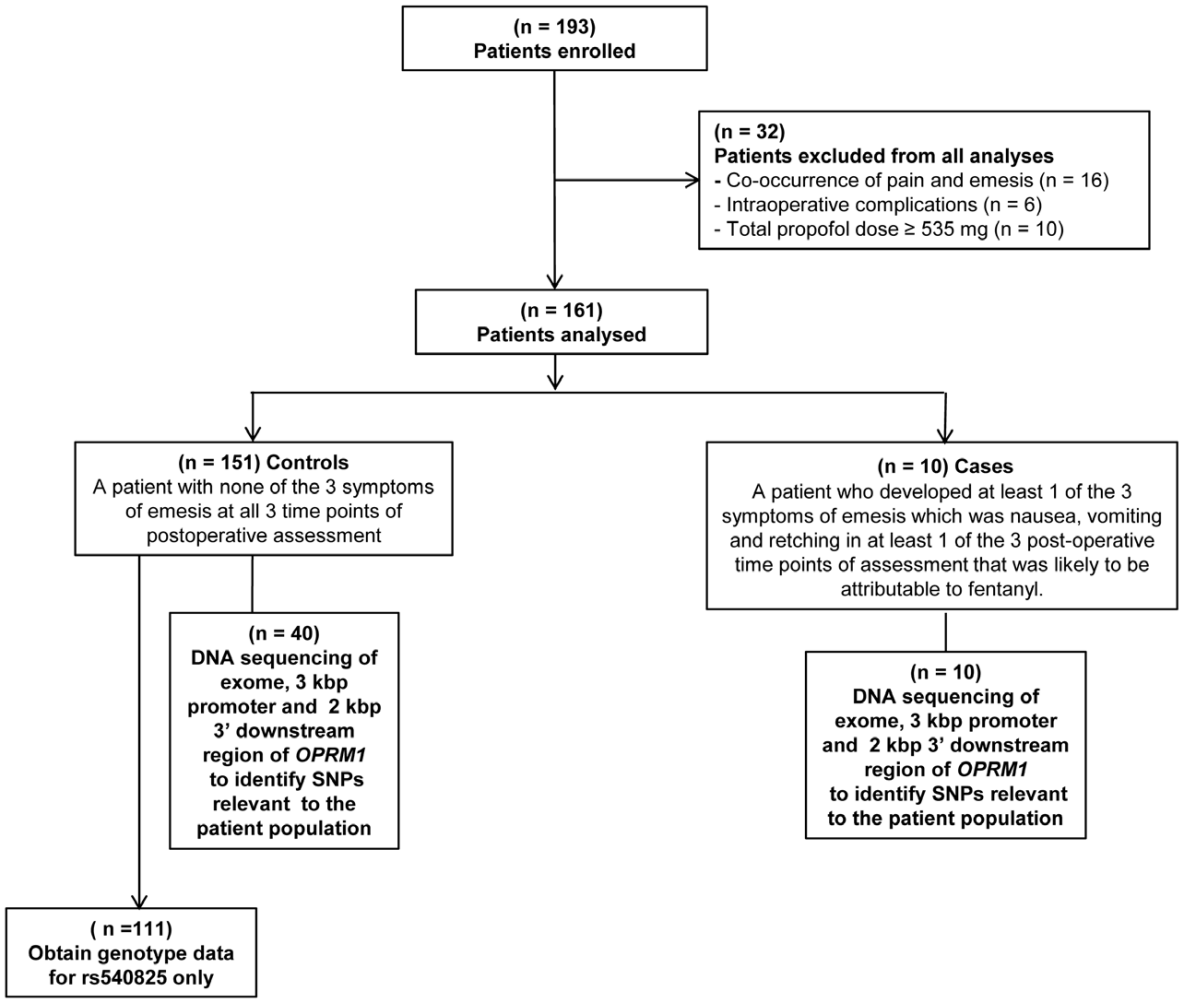
Flowchart outlining the patients enrolled, analysed, classified as cases or controls and sequenced for *OPRM1* SNPs.

Altogether, 193 patients were recruited in this study. However, 32 out of 193 patients were excluded from further analyses for the following reasons. Firstly, 16 had concurrent pain and emesis making it unclear if the occurrence of emesis was directly related to fentanyl while 6 developed unforeseen intraoperative complications requiring the administration of medications, such as midazolam, which might have anti-emetic properties. The final 10 patients were outliers for total propofol dose and they may have been inadvertently misclassified as controls because propofol has anti-emetic properties [20]. Hence, only 161 patients comprising 151 controls and 10 cases, were further analyzed (Figure 2).

A case was defined as a patient who developed a minimum of 1 out of 3 symptoms of emesis that was likely to be fentanyl-related; namely, nausea, vomiting and retching, in at least 1 of 3 postoperative time points of assessment. A control was defined as a patient who did not develop any of the 3 symptoms of emesis at all 3 time points postoperatively.

Nausea, vomiting and retching was treated as a single variable (emesis) with a dichotomous (absent/present) outcome due to the small number of cases which limits meaningful sub-analyses of the emetic phenotype. The rationale for treating nausea and vomiting as a single phenotype is that there may be overlap between the molecular pathways for fentanyl-related nausea and vomiting even though the physiological pathways for nausea differs from that of vomiting.

### DNA Sequencing

DNA from the venous blood of subjects was extracted using the QIAamp DNA Mini Kit (Qiagen, USA).

To identify SNPs relevant to the study population, sequencing of these *OPRM1* regions was initially undertaken in all 10 cases and 40 controls selected by a technician without preference for any specific sample. A control∶case ratio of 4∶1 was used as the basis for sequencing 40 controls and 10 cases as increasing the control∶case ratio beyond 4 to 1 was reported to yield only marginal increases in study power [21].

Polymerase chain reaction (PCR) and sequencing primers were designed to cover the following genomic regions:

- 3 kbp upstream from the start of main exon 1 (promoter).
- 5′ and 3′ untranslated (UTR) regions.
- All 4 main exons (including at least 100 bp at the exon-intron junctions) coding for the major MOR transcript (MOR1).
- 14 alternative exons belonging to 12 human MOR splice variants as reported in the National Centre For Biotechnology Information (NCBI) database.
- 2 kbp of the 3′downstream region from the end of the last (4^th^) main exon of *OPRM1*.

To ensure full sequencing coverage of the promoter and 3′ downstream regions, the sequencing primers span a mean overlap of 145 nucleotides between 2 sequenced regions (Figures S1 and S2 in the Supporting Information). The identified genomic regions in *OPRM1* were amplified by PCR using 10 ng of sample DNA, 5 µl of PCR mastermix from the Qiagen Multiplex PCR kit (Qiagen, USA) and 0.2 pmol/ul of forward and reverse primers in a 10 µl reaction volume. 10 µl of the PCR product was treated with 1 µl ExoSAP (Exonuclease I (Exo) (New England Biolabs, USA):shrimp alkaline phosphatase (SAP) (Promega, USA) at a ratio of 1∶10) at 37°C for 1 hr and 80°C for 15 minutes to remove unincorporated nucleotides and primers.

Big Dye sequencing reactions were performed using 1 µl of ExoSAP treated PCR product, 1 µl of Big Dye Terminator v3.1 Cycle Sequencing Kit (Applied Biosystems (ABI), Life Technologies, USA) and sequencing primer with a final primer concentration of 0.16 pmol/ul in a 10 µl sequencing reaction volume. DNA was precipitated with 100% ethanol and rinsed with 70% ethanol before resuspension of the DNA pellet in 10 µl of Hi-Di solution (ABI, Life Technologies, USA). The DNA was then sequenced using the Genetic Analyzer 3100XL (ABI, Life Technologies, USA). Details regarding all primers, PCR and sequencing thermocycling conditions are listed in Tables S1 and S2 of the Supporting Information.

Base-calling of peaks in the chromatogram was performed using Sequencing Analysis Software v5.3.1 (ABI, Life Technologies, USA) and ContigExpress in Vector NTI ® Advanced 11 (Invitrogen, USA) with corresponding manual chromatogram checks. Double peaks at the same position in the chromatogram that have not been previously identified as SNP loci in NCBI or SNP loci that deviated from Hardy-Weinberg Equilibrium (HWE) underwent bidirectional sequencing to confirm the identity of the peaks.

As rs540825 was noted to be significantly associated with FIE after the initial sequencing, the alternative exon containing this SNP was sequenced in the remaining 111 controls to obtain genotype information for this SNP.

Information for 4 novel SNPs identified during sequencing was deposited in NCBI dbSNP. The accession numbers (ss numbers) for novel variants 1, 2, 3 and 4 in Table 2 are ss528308597, ss 528308608, ss 528308611 and ss 528308612 respectively.

**Table 2:** 25 polymorphic SNPs identified during DNA sequencing of 40 controls and 10 cases.

	Gene Loci					Genotype Frequency In Our Study (%)			CA Trend		Fishers-Exact
	According To	rs	MOR	AA		Homozygous		Homozygous	Test		Test
SNP	MOR1^1^	Number	Transcript	Change	Patients	Major^3^	Heterozygote^3^	Minor^3^	p-value^4^	MAF	p-value^4^
1	5′UR/T-2694**G** 2	rs12210856	-		Cases	90.00	10.00	-	0.197	5.00	0.46
					Controls	70.00	30.00	-		15.00	
2	5′UR/A-2455**C**	rs12190259	-		Cases	90.00	10.00	-	0.197	5.00	0.46
					Controls	70.00	30.00	-		15.00	
3	5′UR/G-1509**A**	rs12205732	-		Cases	90.00	10.00	-	0.197	5.00	0.46
					Controls	70.00	30.00	-		15.00	
4	5′UTR/G-172**T**	rs6912029	MOR1		Cases	90.00	10.00	-	0.156	5.00	0.29
					Controls	67.50	32.50	-		16.25	
5	E1/A118**G**	rs1799971	MOR1	Asn→Asp	Cases	30.00	70.00	-	0.643	35.00	0.80
					Controls	32.50	55.00	12.50		40.00	
6	I1/G-2994**A**	rs563649	MOR1K1		Cases	90.00	10.00	-	0.247	5.00	0.45
			5′UTR		Controls	72.50	27.50	-		13.75	
7	I1/G -2259**A**	rs9322446	MOR1K1		Cases	90.00	10.00	-	1.000	5.00	1.00
			5′UTR		Controls	90.00	10.00	-		5.00	
8	E3/G877**A**	NV1	MOR1	Val**→**Ile	Cases	100.00	0.00	-	0.471	0.00	1.00
					Controls	95.00	5.00	-		2.50	
9	I3/G399**T**	NV2	MOR1A		Cases	90.00	10.00	-	0.043	5.00	0.20
			3′UTR		Controls	100.00	0.00	-		0.00	
**10**	I3/A1839**T**	**rs540825**	**MOR1X**	**Glu→His**	**Cases**	**60.00**	**40.00**	**-**	**0.002** * **(0.035** * **)**	**20.00**	**0.01** * **(0.15)**
			**E4**		**Controls**	**95.00**	**5.00**	**-**		**2.50**	
11	I3/C1956**T**	rs675026	MOR1X	Gly**→**Gly	Cases	60.00	30.00	10.00	0.030* (0.456)	25.00	0.04* (0.60)
			E4		Controls	87.50	10.00	2.50		7.50	
12	I3/A1966**G**	rs562859	MOR1X	Leu**→**Leu	Cases	70.00	20.00	10.00	0.103	20.00	0.11
			E4		Controls	87.50	10.00	2.50		7.50	
13	I3/C-11152**T**	NV3	MOR1B5	Glu**→**Stop	Cases	60.00	40.00	-	0.042* (0.633)	20.00	0.05
					Controls	92.50	5.00	2.50		5.00	
14	I3/C18957**T**	rs606545	MOR1B1		Cases	60.00	40.00	-	0.042* (0.633)	20.00	0.05
			3′UTR		Controls	92.50	5.00	2.50		5.00	
15	3′UTR/A725**G**	rs17181352	MOR1		Cases	100.00	0.00	-	0.614	0.00	1.00
					Controls	97.50	2.50	-		1.25	
16	3′DR/A148**G**	rs671531	-		Cases	80.00	20.00	-	0.459	10.00	0.60
					Controls	92.50	5.00	2.50		5.00	
17	3′DR/A633**G**	rs583664	-		Cases	70.00	30.00	-	0.158	15.00	0.14
					Controls	92.50	5.00	2.50		5.00	
18	3′DR/C932**T**	rs658156	-		Cases	60.00	40.00	-	0.042* (0.633)	20.00	0.05
					Controls	92.50	5.00	2.50		5.00	
19	3′DR/G971**A**	rs9371776	-		Cases	100.00	-	-	0.372	-	1.00
					Controls	92.50	7.50	-		3.75	
20	3′DR/G1270**A**	rs558948	-		Cases	60.00	40.00	-	0.042* (0.633)	20.00	0.05
					Controls	92.50	5.00	2.50		5.00	
21	3′DR/T1371**C**	rs558025	-		Cases	60.00	40.00	-	0.042* (0.633)	20.00	0.05
					Controls	92.50	5.00	2.50		5.00	
22	3′DR/C1510**T**	rs645027	-		Cases	80.00	20.00	-	0.44	10.00	0.73
					Controls	67.50	32.50	-		16.25	
23	3′DR/C1549**T**	rs598160	-		Cases	60.00	40.00	-	0.042* (0.633)	20.00	0.05
					Controls	92.50	5.00	2.50		5.00	
24	3′DR/C1657**G**	rs644261	-		Cases	60.00	40.00	-	0.042* (0.633)	20.00	0.05
					Controls	92.50	5.00	2.50		5.00	
25	3′DR/G1665**A**	NV4	-		Cases	100.00	-	-	0.614	-	1.00
					Controls	97.50	2.50	-		1.25	

Footnote: SNP 10, rs540825, was significantly associated with fentanyl-induced emesis pre- and post- Bonferroni correction.

Abbreviations: SNP: Single nucleotide polymorphism; UR: Upstream region; DR: Downstream region;

UTR: Untranslated region; E: Exon; I: Intron; NV: Novel variant; MOR: Mu opioid receptor;

NCBI: National Centre For Biotechnology Information; CA: Cochran-Armitage; AA: Amino acid;

MAF: Minor allele frequency; Asn: Asparigine; Asp: Aspartate; Val: Valine; Ile: Isoleucine;

Glu: Glutamine; His: Histidine; Gly: Glycine; Leu: Leucine.

Legends:

1 MOR1 is the main transcript of OPRM1. Other transcripts of MOR1 e.g. MOR1X and MOR1K1 are splice variants of MOR.

2 The letter in capitals refers to the minor allele of the SNP.

3 Patients with 2 copies of the major or minor allele respectively. Heterozygote: Patients with 1 copy each of the major and minor allele.

4 Non-bracketed p-values for the CA trend test and Fishers-Exact test refers to p-values before Bonferroni correction. Bracketed (p-values) refers to p-values post-Bonferroni correction.

* p-value<0.05 indicates statistical significance.

### Statistical Analyses

#### Clinical Data

Incidence of FIE was calculated as the total number of patients (n = 10), who developed emesis that was likely to be fentanyl-related divided by the total number of patients who were at risk of developing FIE (n = 161). Outliers for total propofol dose (TPD≥535 mg) were determined based on the following formula:
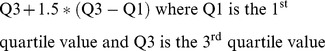



These outliers were excluded from further analyses since propofol has anti-emetic properties and the patients could have been misclassified [20]. The median was used to compare age, total propofol and total fentanyl dose between cases and controls. The statistical significance of differences in median between the 2 groups was determined using the Mann-Whitney test. All basic statistical analyses of the clinical data was performed using Minitab®15 (Minitab Inc,USA).

#### Genotype Data

HWE was calculated for each SNP in the controls. SNPs with HWE<0.001, indicating significant deviation from HWE, were discarded from analyses [22] due to possible errors in base-calling for such SNPs. SNP allele and genotype frequencies were calculated. The Fishers-Exact test was used to test for the significance of allelic frequency differences between cases and controls. The Cochran-Armitage (CA) Trend test for genotypes was used to correlate SNP genotypes with FIE under a co-dominant model [23]. Haplotypes of SNPs for each identified sequenced genomic region and their frequencies were inferred using the Expectation-Maximization (EM) algorithm [24]. PLINK, an online tool for genetic association analyses [25], was used to perform the CA Trend test and haplotype analysis.

Linkage disequilibrium (LD) between 2 SNPs was represented by the pairwise r^2^ association, where r^2^≥0.8 indicates high LD suggesting strong association. The LD profile surrounding the SNP of interest was determined in our study population and compared with other HAPMAP populations including CHB (Han Chinese in Beijing) and CEU (Utah residents with Northern and Western European ancestry) [26].

Bonferroni multiple test correction was performed to correct for Type I error. The uncorrected p-values were multiplied by a factor of 15, which represents the number of SNPs whose occurrence was considered to be independent of other SNPs. The number of independent SNPs was derived from the LD profile of the sequenced SNPs where 14 out of 25 SNPs could be clustered into 4 groups (Figure S3 of the Supporting Information).

The odds ratio (OR) for SNP-phenotype correlations was calculated as the ratio of the odds of developing FIE (cases) to the odds of not developing FIE (controls) with the SNP allele. The population attributable risk (PAR) is the proportion of patients in our study with a particular allele, genotype or haplotype that significantly correlate with FIE. The PAR was calculated using the formula PAR = P*_e_*(R-1)/1+P*_e_*(R-1) where R is the relative risk and P*_e_* is the proportion of patients with the allele, genotype or haplotype that significantly correlates with FIE [27].

Logistic regression for a binary outcome (FIE) incorporating the variables of age, total propofol dose, total fentanyl dose, type of surgery (dilation/curettage/hysteroscopy versus other types of minor gynaecological surgery) and SNP genotype was performed for 151 controls and 10 cases using Minitab®15 (Minitab Inc,USA).

## Results

Ten out of 161 patients analysed in this study developed emesis, which was likely to be fentanyl-induced, giving an FIE incidence of 6.2%.

Three variables that may have contributed to differences in emetic occurrence between cases and controls were total propofol dose, total fentanyl dose and age. Total fentanyl and total propofol dose can differ between individuals as these variables are dependent on the patients' weight and age in this study although the fentanyl dose (µg/kg) and the perioperative propofol plasma concentration (µg/ml) is similar amongst all the patients.

**Figure 3:**
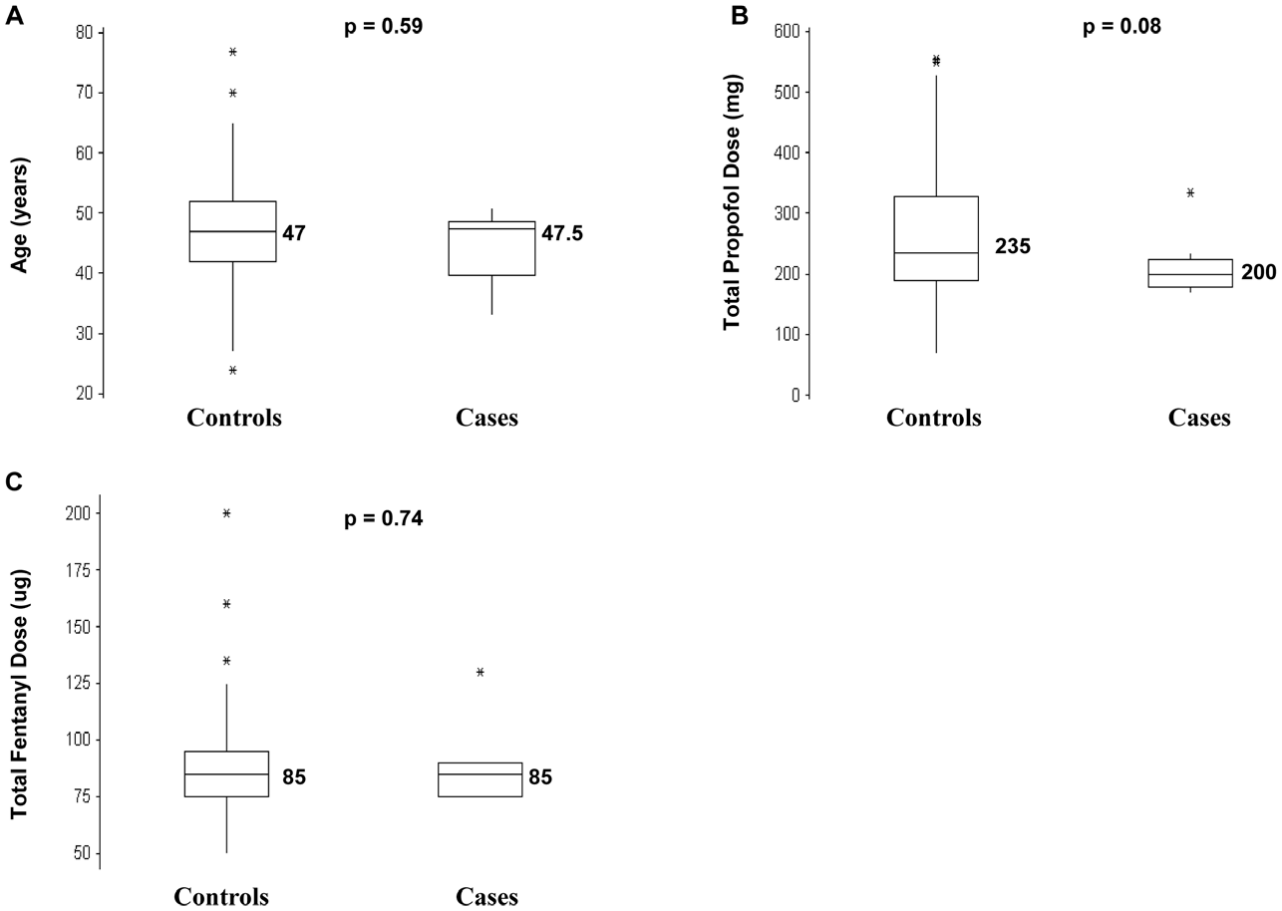
Boxplots indicating the distribution of values for 3 variables in 151 controls and 10 cases. These 3 variables could contribute to the occurrence of emesis but could not be controlled for in the study design (a) Age (b) Total propofol dose (c) Total fentanyl dose. The median is the horizontal line bisecting the shaded box of the boxplot and the median value placed adjacent to the horizontal line. There are no significant differences (p<0.05) between the median values of controls and cases for the 3 variables.

However, no significant differences in the median values for these 3 variables between cases and controls were observed (Figure 3A–C). Hence, these 3 variables were unlikely to contribute significantly to differences in the occurrence of postoperative emesis.

Altogether, 25 polymorphic SNPs were identified in the sequenced regions of 40 controls and 10 cases (Table 2).

### rs540825 is a non-synonymous SNP in an alternative exon of *OPRM1* that significantly correlates with FIE

Amongst the 25 SNPs, there was a significant difference (p = 0.01) before Bonferroni correction in the minor allele frequency (MAF) of SNP rs540825 between 40 controls and 10 cases (Table 2). Notably, the CA Trend test [23] for genotypes showed a significant correlation between the genotypes of rs540825 and FIE both pre (p = 0.002) as well as post-Bonferroni (p = 0.035) correction in 40 controls and 10 cases (Table 2).

To determine if the significant correlation would still be observed when more samples were examined, the exon containing this SNP was sequenced in an additional 111 controls since there were no additional cases. Notably, results similar to that observed with the smaller sample size were obtained when more controls were examined (Fishers-Exact test (p = 0.04 (pre-) and p = 0.60 (post-Bonferroni)) as well as the CA Trend test (p = 0.001 (pre-) and p = 0.019 (post-Bonferroni)).

The OR in 151 controls and 10 cases was 5.6 (95% CI: 1.42–21.91) suggesting that individuals carrying this SNP were 5.6 times more likely to develop FIE. In addition, the PAR analyses indicated that 31.2% of FIE in our study could be attributed to the minor allele of rs540825.

Logistic regression of the 5 variables listed in the Materials and Methods, that could contribute to the emesis in this study, indicated that the rs540825 genotype was the only variable that significantly affected the risk of developing FIE (OR: 3.96, 95%CI: 1.12–14.08). The OR for other non-genetic variables such as surgery and total propofol dose ranged from 0.97 to 1.05.

This SNP was observed to be in low linkage disequilibrium (LD) (r^2^<0.8) with other sequenced polymorphic SNPs in our study (Figure S3 of the Supporting Information). Moreover, its LD pattern in this study concurred with its LD pattern in the CHB and JPT populations from HAPMAP [28] suggesting that this was highly likely to be the SNP contributing to FIE.

## Discussion

The 2 main findings in this study were a low incidence of FIE under the specific study conditions and the significant correlation of FIE with a non-synonymous SNP, rs540825. This SNP resides in the alternative last exon encoding the C-terminus of MOR1X, a known splice variant of MOR, which is differentially expressed within the brain [29].

Exonic SNPs encoding the main transcript have been reported to be responsible for inter-individual differences in drug response [1], [30], [31]. Our study highlights that non-synonymous SNPs, in alternative exons of splice variants, represent another important genetic source of variation in drug response.

The effect size of this SNP is comparable to the effect sizes for ADRs in other reports where the OR ranges from 2.2 (HLA-A*020 allele in drug induced liver injury) [32] to 9 (rs1805128 allele in *KCNE1* for drug-induced Torsades de Pointes) [33] and >20 (HLA-A*3101 allele in Stevens–Johnson syndrome and toxic epidermal necrolysis) [34]. The OR observed in our study also reflects our study design where extreme phenotypes occur in small numbers but the genetic basis for the phenotype is strong. The PAR of this SNP (31.2%) is also notably higher than the PAR of other single SNPs implicated in drug response. For example, the PAR of 2 SNPs implicated in thiazolidinedione-related odema was 29.8% and 18.8%, [35] whilst the PAR of the rs7158782 SNP in *TCL1A*, which conferred an increased risk of aromatase inhibitor-related musculoskeletal side effects, was 11% [36], [37].

Interestingly, the MAF of this SNP in the HAPMAP Caucasian (CEU 27.9%) and the Chinese (Chinese in Singapore (CHS) 6.5%, CHB 4.2%, CEU 27.9%) populations is very different, suggesting that the effect of this SNP on FIE risk may be population specific. Population-specific effects of SNPs have been previously reported for ADRs [38]. Significantly, when F_ST_, which measures the degree of genetic differentiation between 2 populations, was calculated for this SNP using data from the HAPMAP Public Release #27 dataset, the F_ST_ for the CHB-CEU populations (F_ST (CHB-CEU)_) was 0.17, placing it amongst the top 5% of F_st_ values for all SNPs in the CHB-CEU populations (unpublished data courtesy of M. Bachtiar).

This SNP has also previously been associated with a specific subset of citalopram (antidepressant) response that was observed only in non-Hispanic-Whites but not African-Americans or Hispanic-Whites [39]. In contrast, Laugsand *et al*, reported no significant correlation between this SNP and OIE in a study of 1579 European patients with advanced cancer taking opioids for symptom control, where 96 SNPs with MAF≥10% were genotyped [40]. A plausible explanation for the disparate findings may lie in the different study design employed by the European investigators and us.

In the European study, subjects were heterogeneous for at least 10 variables that may act as confounders for the genotyped SNP and OIE. These confounders included the age, type of cancer, site of metastases, age, gender, type of opioid, stability of opioid dosing, concomitant use of anti-emetics, steroids in the past 24 hours in addition to other medications, past medical history and clinical setting (outpatients or hospital inpatient). The large number of confounders creates difficulty in distinguishing emesis due to opioids from non-opioid causes as well as genetic from non-genetic factors. Notably, if the European study was conducted in populations of Chinese descent where the MAF of this SNP is <10%, this SNP would not have been genotyped.

This SNP, rs540825, changes an amino acid in the C-terminus of the splice variant, MOR1X, from a neutral (glutamine) to basic (histidine) amino acid. MOR1X was previously reported to be functionally different from MOR1, the main transcript [29]. In addition, mutations in the C-terminus of MOR1 have been reported to affect agonist potency and affinity [41], [42]. Hence, this SNP, which results in a non-synonymous change at the C-terminus of MOR1X, could conceivably affect MOR1X structure and function. Although Garriock *et al* [39] did not observe significant differences between the major and minor alleles of this SNP for adenylyl cyclase inhibition and ligand-induced receptor endocytocis *in vitro*, the functional effect of the minor allele may be related to established MOR signaling processes such as ion channel coupling, protein kinase phosphorylation [41] and MOR dimerization with other receptors [43], which do not involve adenylyl cyclase/cAMP and were not investigated in their study.

The main limitations of this study are the lack of a second study to demonstrate the replication of the association of rs540825 with FIE and the small case numbers. Replication studies may be the norm for genetic association studies of common disease. However, undertaking replication studies for rare ADRs, including FIE in our study, or extreme phenotypes, which tend to be few in number, can be daunting due to the difficulties in collecting large numbers of cases over a reasonable period of time [44]. The complexities of emesis also make subgroup analyses difficult due to the small numbers in each subgroup.

This study describes the first association between a non-synonymous SNP in an alternative splice variant of OPRM1, rs540825, and FIE. Our study finding could potentially contribute to the development of future preoperative screening tools to identify individuals at risk of FIE for prophylactic anti-emetics. However, further studies are needed to evaluate this association in different ethnic groups and confirm the clinical utility of predicting the FIE risk.

## Supporting Information

Figure S1 **Regions of OPRM1 amplified by PCR and sequenced.** (a) 3 kbp of 5′ upstream region (promoter) (b) Main coding exons (c) 2 kbp downstream of the last main exon of *OPRM1*. Sequencing primers used are listed in Tables S1 and S2 of the Supporting Information. The mean number of nucleotides overlapping between sequenced regions = 145 bp. **Abbreviations:** PCR: Polymerase chain reaction PF: forward primer for PCR PR: reverse primer for PCR.
(PDF)

Figure S2 **Amplified and sequenced alternative exons (AE) in** ***OPRM1*** **for 12 mu-opioid receptor (MOR) splice variants reported in the National Centre for Biotechnology Information (NCBI) database.** (a) AE's in intron 3 and the region 3′ downstream of *OPRM1* (b) AE's in the 5′ region upstream of *OPRM1* (c) AE's involving >1 region of *OPRM1*. Sequencing primers were located at least 100 bp away from the start and end of the AE. MOR1 is the main transcript of MOR. MOR1 exons were referred to as the main *OPRM1* exons. Other transcripts such as MOR1A and MOR1B4 are referred to as splice variants and AE's are only found in the splice variant transcripts.
(PDF)

Figure S3 **Pairwise linkage disequilibrium (LD) pattern of the following sequenced regions of** ***OPRM1*** **; 3 kbp of the promoter region, exons coding for the main mu-opioid receptor transcript (MOR1), 14 alternative exons of** ***OPRM1*** **that code for 12 mu-opioid receptor splice variants and 2 kbp of the 3′ region downstream of** ***OPRM1*** **.** SNPs with r^2^≥0.8 indicate high LD between the 2 SNPs as reflected by the increased colour intensities in the LD plot. There are 4 groups of SNPs with high LD and 11 SNPs who are not in high LD with other SNPs identified during sequencing. SNP rs540825 (in bold), which was significantly associated with fentanyl-induced emesis, has low LD with other SNPs. Abbreviations: UR: upstream region, UTR: untranslated region NV: novel variant kbp: kilobase pair
(PDF)

Table S1 **Primers used for polymerase chain reaction (PCR) and sequencing 3 kbp upstream of the 1st main exon, all 4 main exons and 2 kbp of the 3′ region downstream of the 4th main exon of OPRM1.**
(XLSX)

Table S2 **Primers used for polymerase chain reaction (PCR) and sequencing OPRM1 alternative exons.**
(XLSX)
