# Supplementary material for: A Non-Synonymous Single Nucleotide Polymorphism in an OPRM1 Splice Variant Is Associated with Fentanyl-Induced Emesis in Women Undergoing Minor Gynaecological Surgery
Source: PLoS One. 2012 Nov 7;7(11):e48416. doi: 10.1371/journal.pone.0048416 (PMC3492352; doi:10.1371/journal.pone.0048416)

Supplementary Figure 1: **Regions of *OPRM1* amplified by PCR and sequenced. (a) 3 kbp of 5' upstream region (promoter) (b) Main coding exons (c) 2 kbp downstream of the last main exon of *OPRM1*. Sequencing primers used are listed in supplementary Table 1 and 2. Mean number of nucleotides overlapping between sequenced regions =145 bp.**
Abbreviations: PCR: Polymerase chain reaction PF: forward primer for PCR PR: reverse primer for PCR.

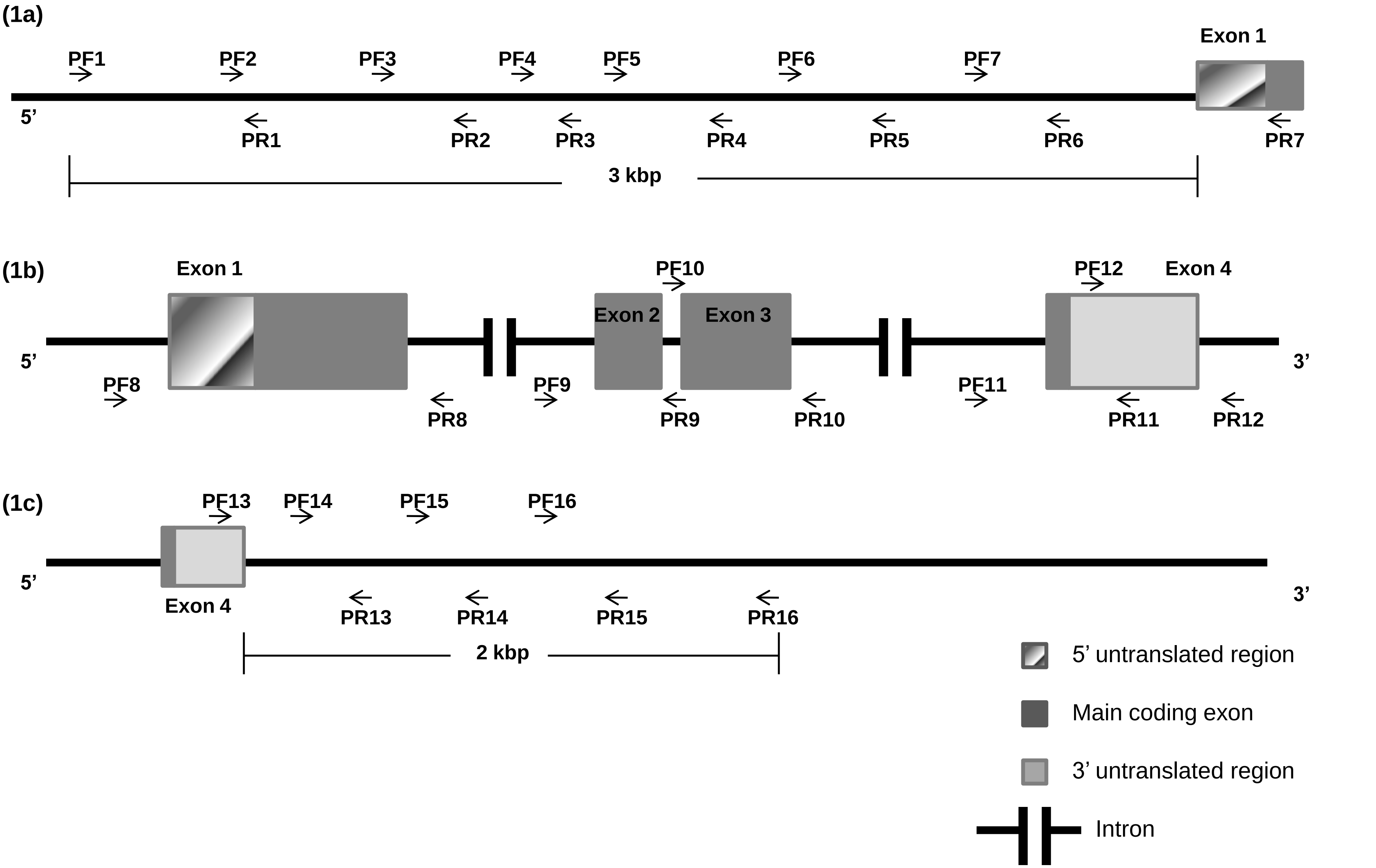

Supplement: Figure S1 — Regions of OPRM1 amplified by PCR and sequenced. (a) 3 kbp of 5′ upstream region (promoter) (b) Main coding exons (c) 2 kbp downstream of the last main exon of OPRM1. Sequencing primers used are listed in Tables S1 and S2 of the Supporting Information. The mean number of nucleotides overlapping between sequenced regions = 145 bp. Abbreviations: PCR: Polymerase chain reaction PF: forward primer for PCR PR: reverse primer for PCR. (PDF) [file pone.0048416.s001.pdf]
