# Supplementary material for: A Non-Synonymous Single Nucleotide Polymorphism in an OPRM1 Splice Variant Is Associated with Fentanyl-Induced Emesis in Women Undergoing Minor Gynaecological Surgery
Source: PLoS One. 2012 Nov 7;7(11):e48416. doi: 10.1371/journal.pone.0048416 (PMC3492352; doi:10.1371/journal.pone.0048416)
Supplement: Figure S2 — Amplified and sequenced alternative exons (AE) in OPRM1 for 12 mu-opioid receptor (MOR) splice variants reported in the National Centre for Biotechnology Information (NCBI) database. (a) AE's in intron 3 and the region 3′ downstream of OPRM1 (b) AE's in the 5′ region upstream of OPRM1 (c) AE's involving >1 region of OPRM1. Sequencing primers were located at least 100 bp away from the start and end of the AE. MOR1 is the main transcript of MOR. MOR1 exons were referred to as the main OPRM1 exons. Other transcripts such as MOR1A and MOR1B4 are referred to as splice variants and AE's are only found in the splice variant transcripts. (PDF) [file pone.0048416.s002.pdf]

<u>Supplementary Figure 2:</u> Amplified and sequenced alternative exons (AE) in *OPRM1* for 12 mu-opioid receptor (MOR) splice variants reported in the National Centre for Biotechnology Information (NCBI) database. (a) AE in intron 3 and the region 3' downstream of *OPRM1* (b) AE in the 5' region upstream of *OPRM1* (c) AE involving >1 region of *OPRM1*. Sequencing primers were located at least 100 bp away from the start and end of the AE. MOR1 is the main transcript of MOR. MOR1 exons were referred to as the main *OPRM1* exons. Other transcripts such as MOR1A and MOR1B4 are referred to as splice variants and AE's are only found in the splice variant transcripts.

(2a)

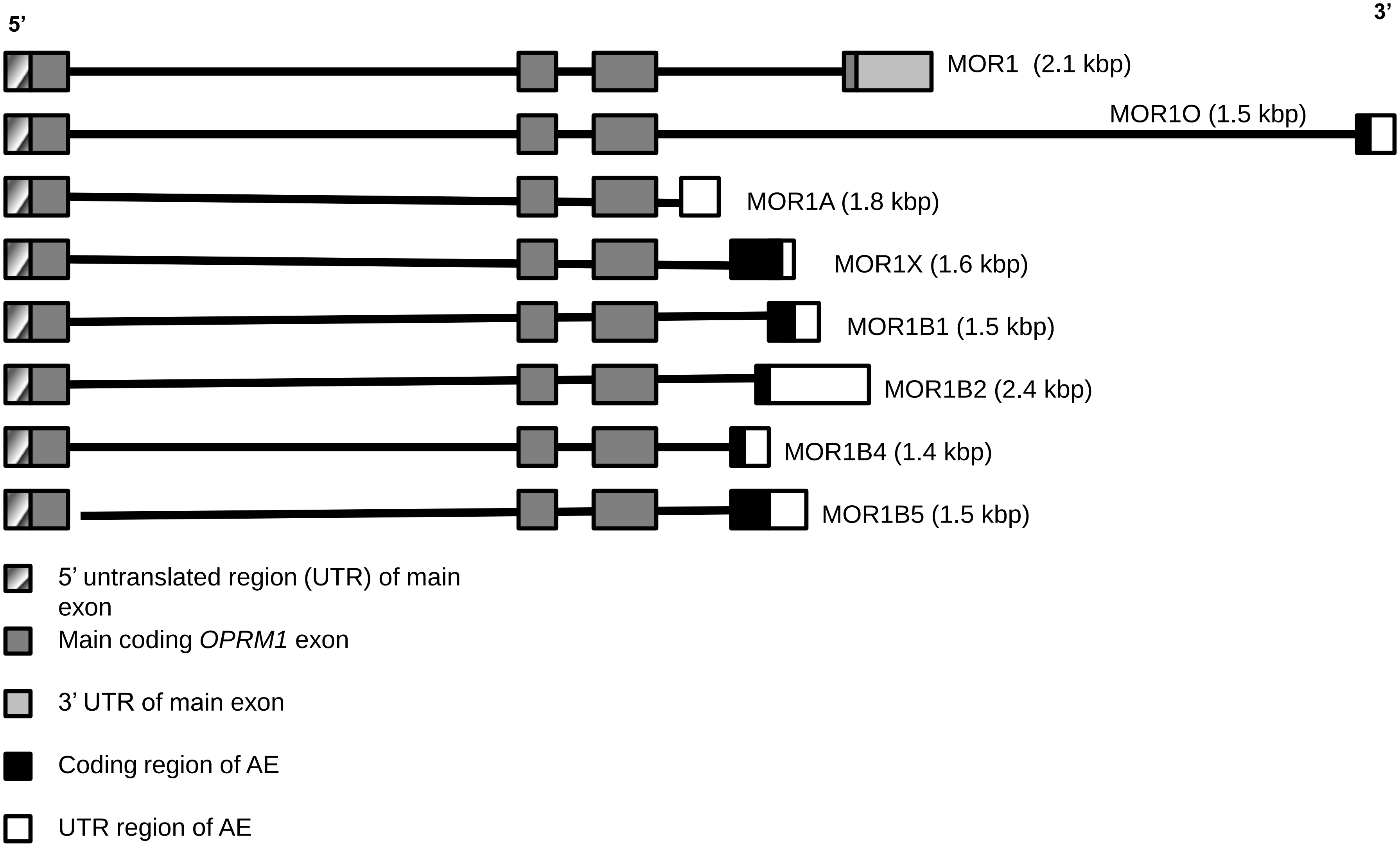

(2b)

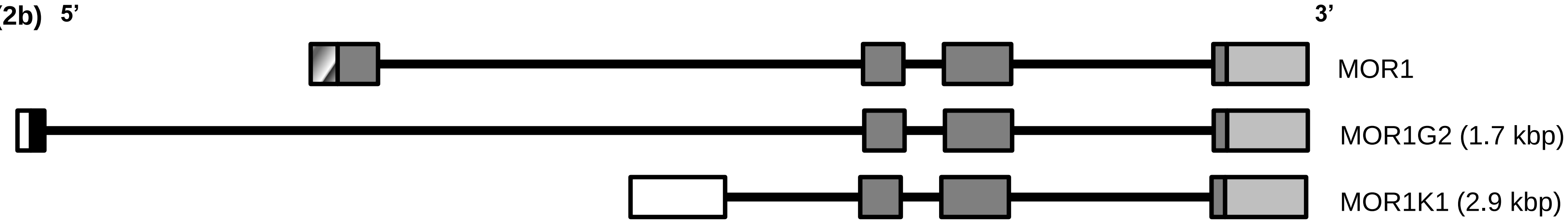
5’
3’
MOR1
MOR1G2 (1.7 kbp)
MOR1K1 (2.9 kbp)

(2c)

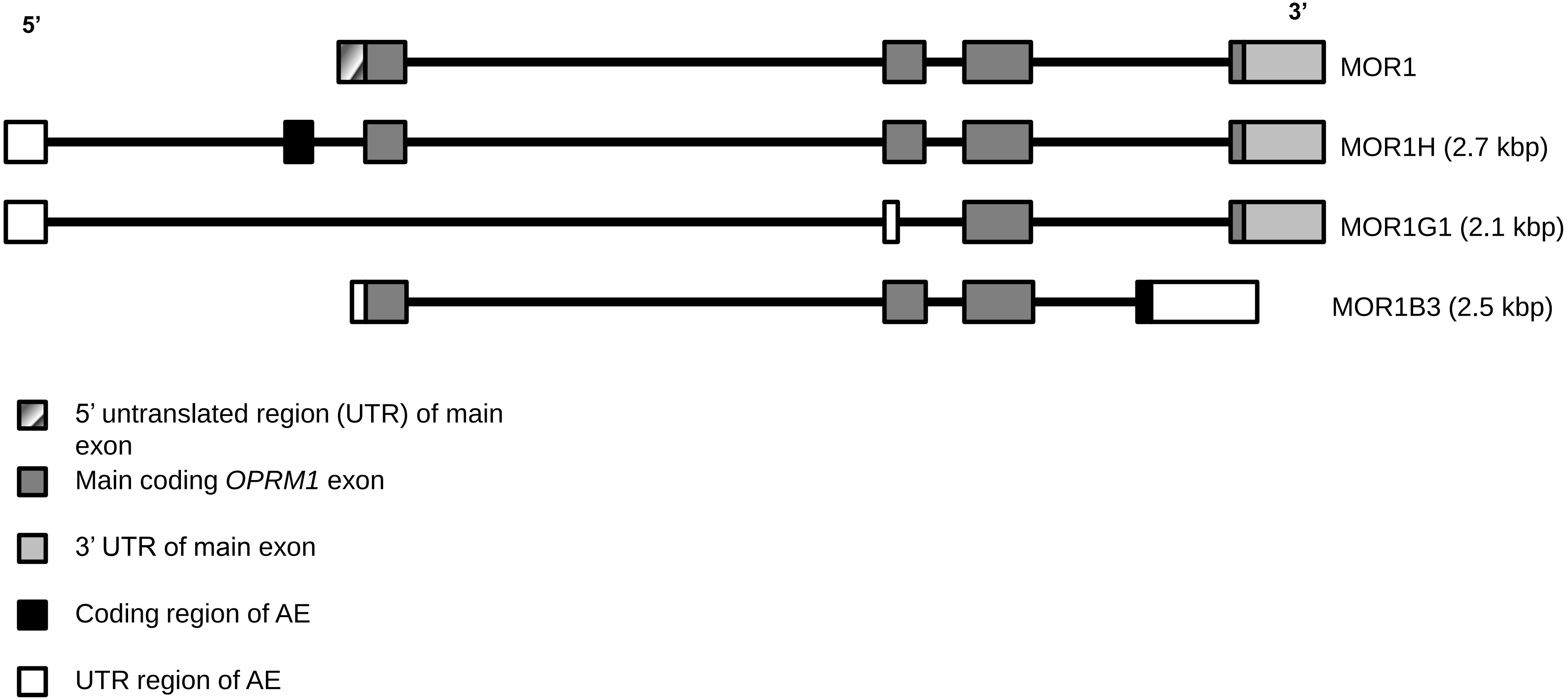
5’
3’
MOR1
MOR1H (2.7 kbp)
MOR1G1 (2.1 kbp)
MOR1B3 (2.5 kbp)
5’ untranslated region (UTR) of main exon
Main coding OPRM1 exon
3’ UTR of main exon
Coding region of AE
UTR region of AE
