# Supplementary material for: A Non-Synonymous Single Nucleotide Polymorphism in an OPRM1 Splice Variant Is Associated with Fentanyl-Induced Emesis in Women Undergoing Minor Gynaecological Surgery
Source: PLoS One. 2012 Nov 7;7(11):e48416. doi: 10.1371/journal.pone.0048416 (PMC3492352; doi:10.1371/journal.pone.0048416)

__Supplementary Figure 3:__ **Pairwise linkage disequilibrium (LD) pattern of the following sequenced regions of *OPRM1*; 3 kbp of the promoter region, exons coding for the main mu-opioid receptor transcript (MOR1), 14 alternative exons of *OPRM1* that code for 12 mu-opioid receptor splice variants and 2 kbp of the 3' region downstream of *OPRM1*. SNPs with $r^2 \geq 0.8$ indicates high LD between the 2 SNPs as reflected by the increased colour intensities in the LD plot. SNP rs540825 (in bold) which was significantly associated with fentanyl induced emesis, has low LD with other SNPs. There are 4 groups of SNPs with high LD and 11 SNPs who are not in high LD with other SNPs identified during sequencing. Abbreviations:**
UR: upstream region, UTR: untranslated region, NV: novel variant kbp: kilobase pair

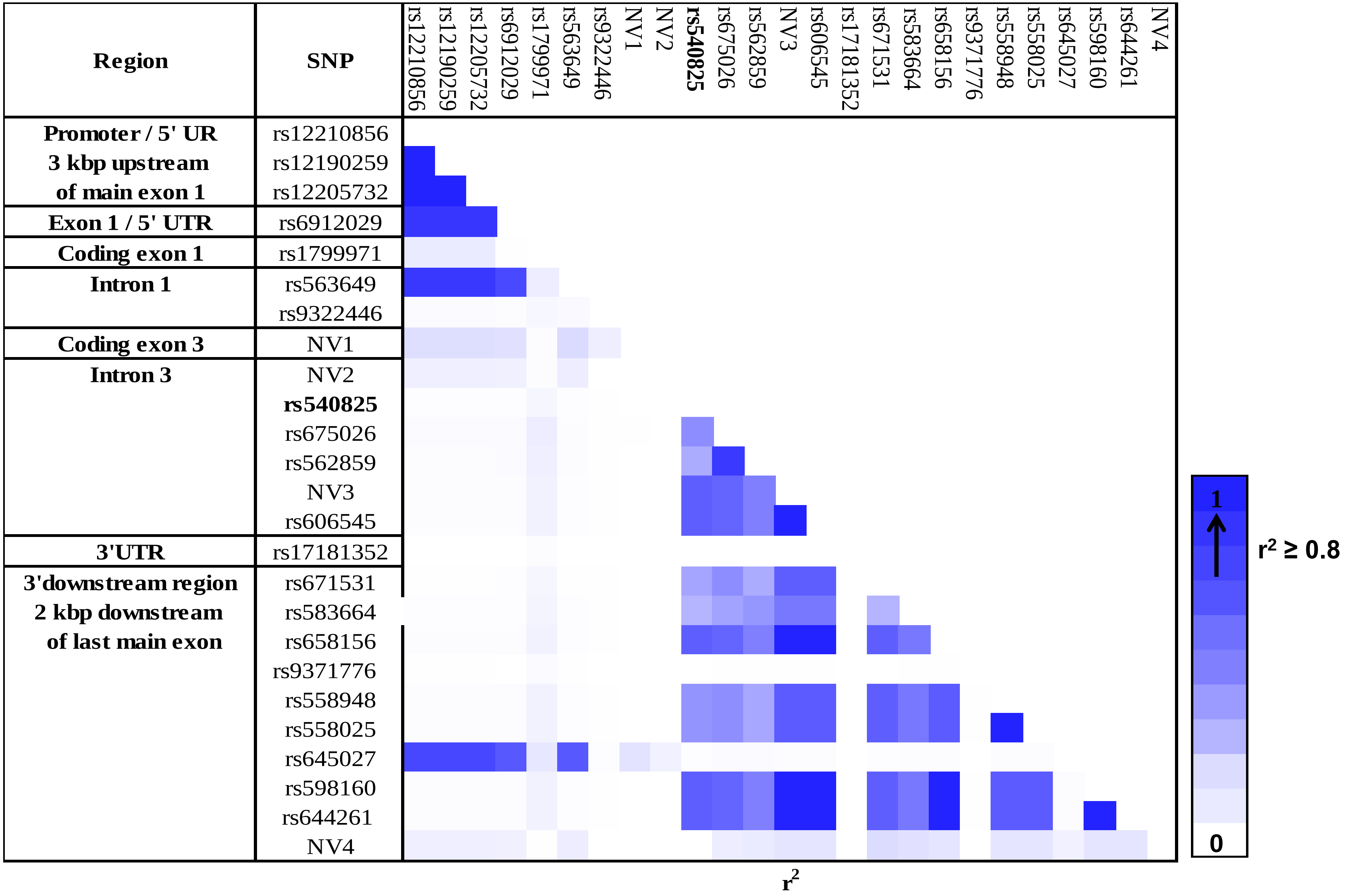

Supplement: Figure S3 — Pairwise linkage disequilibrium (LD) pattern of the following sequenced regions of OPRM1 ; 3 kbp of the promoter region, exons coding for the main mu-opioid receptor transcript (MOR1), 14 alternative exons of OPRM1 that code for 12 mu-opioid receptor splice variants and 2 kbp of the 3′ region downstream of OPRM1 . SNPs with r2≥0.8 indicate high LD between the 2 SNPs as reflected by the increased colour intensities in the LD plot. There are 4 groups of SNPs with high LD and 11 SNPs who are not in high LD with other SNPs identified during sequencing. SNP rs540825 (in bold), which was significantly associated with fentanyl-induced emesis, has low LD with other SNPs. Abbreviations: UR: upstream region, UTR: untranslated region NV: novel variant kbp: kilobase pair (PDF) [file pone.0048416.s003.pdf]
